# Are Anticholinergic Medications Associated With Increased Risk of Dementia and Behavioral and Psychological Symptoms of Dementia? A Nationwide 15-Year Follow-Up Cohort Study in Taiwan

**DOI:** 10.3389/fphar.2020.00030

**Published:** 2020-02-14

**Authors:** Yia-Ping Liu, Wu-Chien Chien, Chi-Hsiang Chung, Hsin-An Chang, Yu-Chen Kao, Nian-Sheng Tzeng

**Affiliations:** ^1^ Department of Psychiatry, Cheng Hsin General Hospital, Taipei, Taiwan; ^2^ Department of Psychiatry, Tri-Service General Hospital, School of Medicine, National Defense Medical Center, Taipei, Taiwan; ^3^ Laboratory of Cognitive Neuroscience, Departments of Physiology and Biophysics, National Defense Medical Center, Taipei, Taiwan; ^4^ Department of Medical Research, Tri-Service General Hospital, National Defense Medical Center, Taipei, Taiwan; ^5^ School of Public Health, National Defense Medical Center, Taipei, Taiwan; ^6^ Graduate of Life Sciences, National Defense Medical Center, Taipei, Taiwan; ^7^ Taiwanese Injury Prevention and Safety Promotion Association, Taipei, Taiwan; ^8^ Student Counseling Center, National Defense Medical Center, Taipei, Taiwan; ^9^ Department of Psychiatry, Tri-Service General Hospital, National Defense Medical Center, Taipei, Taiwan

**Keywords:** anticholinergic, risk, dementia, behavioral and psychological symptoms of dementia, National Health Insurance Research Database

## Abstract

**Background/Objective:**

In previous reports, the usage of anticholinergic medications has been associated with an increased risk of dementia with prolonged usage or with a high Anticholinergic Cognitive Burden (ACB). This study aimed to investigate the association between anticholinergic medications and the risk of dementia using data from Taiwan's National Health Research Database (NHIRD).

**Methods:**

A total of 790,240 patients, with 197,560 patients receiving anticholinergic medications and 592,680 control patients (1:3) matched for sex, age, and index-year, were enrolled from the two million Longitudinal Health Insurance Dataset, a subdataset of the NHIRD, between 2000 and 2015. The time-dependent Cox regression analysis was used to explore the hazard ratio (HR) with a 95% confidence interval for the association between anticholinergics and the risk of dementia during the 15-year follow-up. The behavioral and psychological symptoms of dementia (BPSD) were recognized by the usage of psychotropics. The ACB ranged from zero to three, divided as score <1, 1–1.9, 2–2.9, 3–4.9,and ≧5. The sensitivity analysis was done by excluding the diagnoses of dementia in the first 2 or 4 years after anticholinergic usage.

**Results:**

In the anticholinergic usage cohort, the HR was 1.043 (95% CI = 0.958-1.212, *p* = 0.139) without a significant difference. The sensitivity analysis revealed no association between the usage of anticholinergics and the risk of dementia. Anticholinergic usage was not associated with BPSD. Male sex, patients of ages of 60–64 and ≧80, usage of antiparkinsonian medications, a history of Parkinson's disease, epilepsy, urinary incontinence, depression, bipolar disorder, and psychotic disorder were independent risk factors of dementia. Increased HRs for dementia were associated with an ACB ≥ 5 and an anticholinergic usage period ≥ 1,460 days.

**Conclusion:**

In this study, the usage of anticholinergics was not associated with the risk of dementia or BPSD in a 15-year follow-up study. However, patients with the male sex, patients with ages of 65–79 and ≧80, patients with some comorbidities, high ACB scores, and long anticholinergic treatment duration were associated with the risk of dementia.

## Introduction

Among those age-dependent mental disorders, dementia is probably the most devastating. It marks a broad range of abnormalities across different dimensions of cognitive deterioration and has approximately a 5%–7% lifetime prevalence in the global population (Prince et al., 2013). The prevalence in previous community studies was 4%–8% for the population aged ≥65 years (Liu et al., 1995; Liu et al., 1998a; Liu et al., 1998b; Sun et al., 2014), and dementia could result in a burden for these patients, their caregivers, and the community in Taiwan (Tzeng et al., 2015; Tzeng et al., 2017b; Wang et al., 2018; Yeh et al., 2019).

Behavioral and psychological symptoms of dementia (BPSD) are common in patients with different types of dementia, such as psychosis, agitations, mood disorders, disinhibited behavior, sleep-wake cycle disturbances, wandering, perseveration, pathological collecting, or shouting, which are related to more rapid progression of the disease, earlier institutionalization, use of physical restraints, and higher risk of mortality (Liperoti et al., 2008; Masopust et al., 2018). As the risk factors or leading causes of dementia and the BPSD are multifarious and largely undetermined, the treatment efficacy of this disease is unsatisfactory. Future efforts are therefore required to identify the risk factors and then reduce any possible exposure to those risk factors.

Anticholinergic medications are extensively employed in clinical medicine. They are well known for their cognitive side effects, including drowsiness, delirium, sedation, and memory problems (Ruxton et al., 2015), especially among frail, older patients (Tannenbaum et al., 2012). In patients with subjective cognitive decline or neurocognitive disorders, anticholinergic drugs are associated with functional impairment, cognitive impairment, and behavioral disturbances (Carnahan et al., 2017), while the reduction of the anticholinergic burden during treatment would decrease the BPSD (Dauphinot et al., 2017). Educational programs, such as the “Improving Antipsychotic Appropriateness in Dementia Patients Educational Program” and the “Centers for Medicare and Medicaid Services Partnership to Improve Dementia Care”, were used and became effective in reducing the usage of antipsychotics and anticholinergics and reducing BPSD and delirium for patients in nursing homes (Jaidi et al., 2018).

By a prospective, population-based cohort study performed in an integrated health-care delivery system in the United States, Gray and colleagues (2015) reported that the cumulative usage of strong anticholinergic medications may well be associated with a risk of dementia (Gray et al., 2015). Richardson and colleagues (2018) performed a case-control design study and provided evidence to strengthen the association between some classes of anticholinergic drugs and the incidence of later dementia (Richardson et al., 2018).

However, as to whether anticholinergics were associated with dementia should be cautiously judged as this association may be influenced by several factors. First, a longer duration between the time of anticholinergic drug usage and the diagnosis of dementia was important since the development of most types of dementia is gradual and progressive (American Psychiatric Association, 1994; American Psychiatric Association, 2000). Gray and colleagues (2015) confirmed and justified that the dementia risk from anticholinergics is based on a prospective, population-based cohort study with a mean follow-up time of 7.3 years, which might be not enough for the long-term anticholinergic consequences to progress. Second, they found that higher scores of Anticholinergic Cognitive Burden (ACB) scale might have reflected polypharmacy and greater illness burden, which might be associated with the risk of dementia (Gray et al., 2015). One previous study has shown that anticholinergic polypharmacy with higher ACB score was associated with the anticholinergic-associated dementia (Richardson et al., 2018). Third, the anticholinergic medications might induce BPSD in the patients with dementia (Carnahan et al., 2017), but it is not clear as to whether the usage of anticholinergic medications is associated with the risk of BPSD. Therefore, we conducted this study to clarify this missing information.

In the present study, we used the Taiwan National Health Insurance Research Dataset (NHIRD) to examine whether there is an association between previous anticholinergic usage, late-onset dementia, and BPSD during the longer follow-up time of 15 years.

## Methods

### Data Sources

The Taiwan National Health Insurance (NHI) Program was enacted in 1995. The enrolled participants exceeded 99% of the population and were contracted with 97% of the medical providers (Ho Chan, 2010). The details of this program have been documented in previous studies (Huang et al., 2014; Tang et al., 2015; Yang et al., 2015a; Tzeng et al., 2016; Tzeng et al., 2017a; Tzeng et al., 2017c; Chao et al., 2018; Chu et al., 2018; Tzeng et al., 2018; Tzeng et al., 2019b). The data sources of the present study were two million randomly sampled patients from the Longitudinal Health Insurance Database (LHID), a subset of the NHIRD, over a 15-year period (2000-2015). Since several previous studies have revealed a high accuracy and validity of the diagnoses in the NHIRD (Cheng et al., 2011; Liang et al., 2011; Chou et al., 2013; Hsieh et al., 2015), it is therefore suitable to use the NHIRD to examine the longitudinal association between anticholinergic usage and the potential risk of developing dementia later in life.

The diagnostic coding employed in the present study is in accordance with the International Classification of Disease, 9th revision, Clinical Modification (ICD-9-CM) diagnostic criteria from the NHI Administration (Chinese Hospital Association, 2000). All diagnoses of dementia were made by board-certified psychiatrists or neurologists, according to the Diagnostic and Statistical Manual of Mental Disorders, 4th Edition (DSM-IV) and its Text Revised Edition (DSM-IV-TR) (American Psychiatric Association, 1994; American Psychiatric Association, 2000). To verify the accuracy of the diagnoses, the NHI Administration randomly and regularly reviews the records of one in 100 ambulatory care visits and one in 20 in-patient claims (Ministry of Justice, 2014). In addition, licensed medical records technicians verified the coding before claiming the reimbursements in hospitals and clinics (Tzeng et al., 2016; Tseng et al., 2019). Therefore, while only a small number of validation studies with small sample sizes have been undertaken, they have generally reported positive predictive values of over 70% for various diagnoses, thus, the NHIRD is a large, powerful data source for biomedical research (Lin et al., 2018c).

### Study Design and Sampled Participants

The present study was designed as a nationwide, matched cohort study. A total of 790,240 patients, with 197,560 anticholinergic patients and 592,680 control patients. To avoid the survival bias as possible, we used the CICR analysis. The definition of exposed group was that the patients who first started to use anticholinergics in the study period, between January 1, 2000 and December 31, 2015. The matched control, or unexposed group, was 1:3 sex, age, and index-year matched individuals. Both exposed and unexposed groups were enrolled from the 2 million LHID. The anticholinergic exposed group, or the users, were individuals who ever used an anticholinergic during the whole 15-year period. The unexposed (control) group, or nonusers, individual who never used an anticholinergic during the 15-year period. The subjects with dementia or anticholinergic usage before 2000, becoming control, or the entry date were excluded in this study.

**Figure 1:**
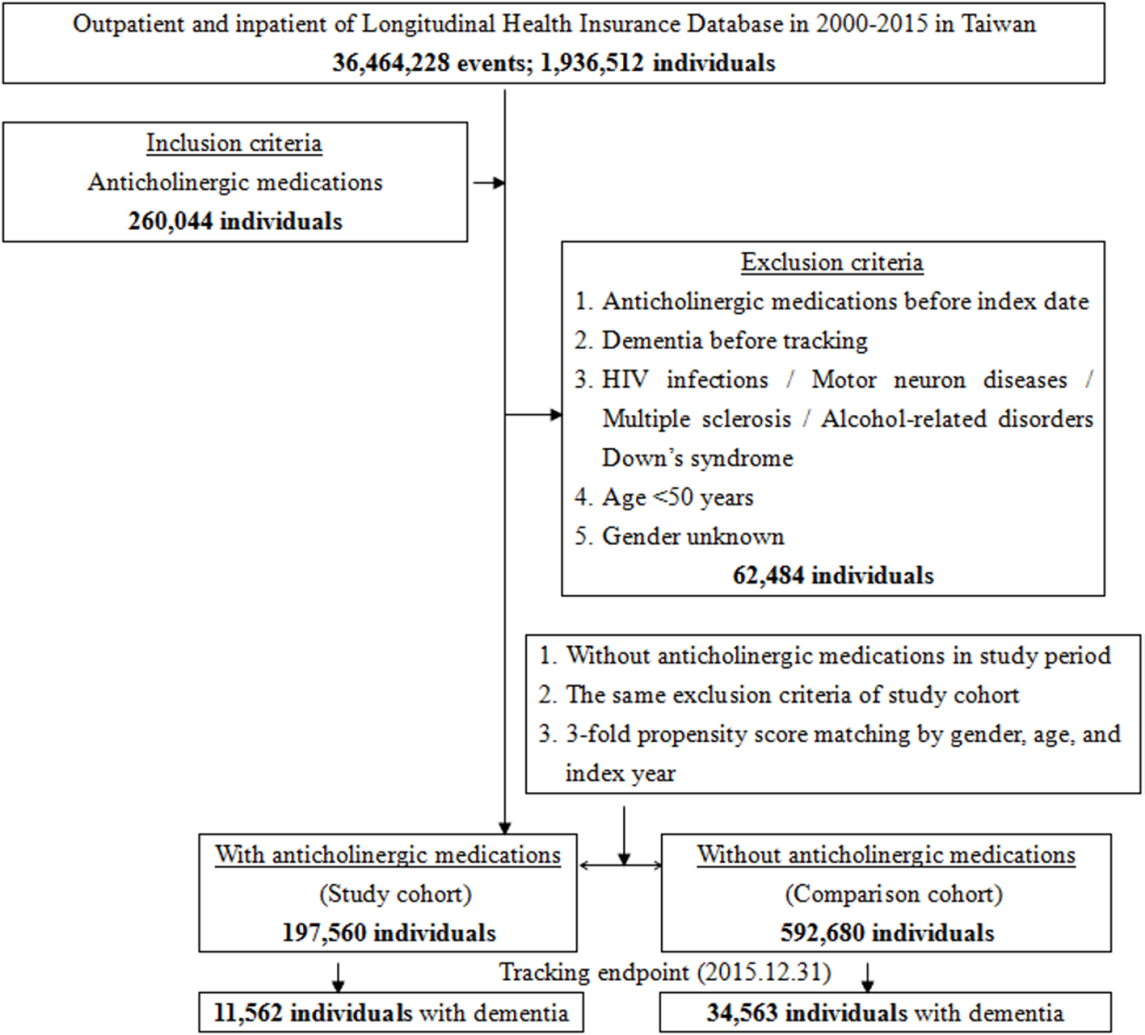
The flowchart of study sample selection from National Health Insurance Research Database in Taiwan.

Anticholinergic drugs are classified into several categories according to the clinical effects: antidepressants, antipsychotic, antiparkinsonian, analgesics, cardiovascular, gastrointestinal, respiratory, urological, and other anticholinergic drugs (Gray et al., 2015; Richardson et al., 2018). The exclusion criteria were those using anticholinergics before 2000, those aged <50 years, and those diagnosed with dementia before January 1, 2000 or their cohort entry date. Patients with one of the following diagnoses before January 1, 2000 were excluded from the present study, including HIV infections (ICD-9 codes of 042, 043, 044, V08), motor neuron diseases (ICD-9-CM 335), multiple sclerosis (340), alcohol-related disorders [ICD-9-CM alcohol-induced psychosis (291.x), alcohol dependence (303.x), alcohol abuse (305.0), alcoholic polyneuropathy (357.5), alcoholic cardiomyopathy (425.5), alcoholic gastritis (535.3), alcoholic liver diseases (571.0, 571.1, 571.2, and 571.3)], Down syndrome (ICD-9-CM 758.0), and dementia (ICD-9-CM 290.0, 290.10, 290.11, 290.12, 290.13, 290.20, 290.21, 290.3, 290.41, 290.42, 290.43, 290.8, 290.9, and 331.0), with references from the two aforementioned studies (**Figure 1**) (Gray et al., 2015; Richardson et al., 2018).

In addition, we included the antipsychotics, antidepressants and sedative-hypnotics of estimated treatment duration ≧30 days, to represent the occurrence of BPSD in both cohorts. We have excluded the mental health conditions before dementia diagnoses that might prompt continued use of psychotropics, such as schizophrenia or bipolar disorder.

### The Classification of Anticholinergic Medications

The anticholinergic medications could be categorized as the following types of drugs: antidepressants, antipsychotics, antiparkinsonian drugs, analgesics, cardiovascular, gastrointestinal (anti-spasmodic and anti-ulcer), respiratory (anti-asthmatics), urological (for urinary incontinence), and other anticholinergics (Gray et al., 2015; Richardson et al., 2018). The present study used the Anticholinergic Cognitive Burden (ACB) as an index to quantify the degree of the anticholinergic exposure by the same score employed by others (Campbell et al., 2013): A score of 1 refers to drugs without the clinically relevant negative cognitive effects. A score of 2 refers to drugs with established and clinically relevant anticholinergic effects. A score of 3 refers to drugs with a score of 2 and are reported to be associated with delirium. All other drugs were scored as 0 (Salahudeen et al., 2015). In addition, the cumulative dosage of the anticholinergic medications was calculated as the sum of the dispensed doses of any anticholinergics from the LHID.

### The Calculation of Duration and Cumulative Doses of the Medications

The data of the defined daily dosage (DDD) were obtained from the WHO Collaborating Center for Drug Statistics Methodology 2018 (https://www.whocc.no/) (World Health Organization, 2018), and the duration of the usage of anticholinergics, antidepressants, and hypnotics was calculated by dividing the cumulative doses by the DDD of these medications. For the usage of antipsychotics in the treatment of BPSD, the average daily doses of haloperidol (1.0 mg a day), risperidone (1.0 mg a day), olanzapine (7.5 mg a day), quetiapine (75 mg a day), and aripiprazole (10 mg a day), were used to calculate the duration of the antipsychotic usage (Chan et al., 2001; Brodaty et al., 2003; Lee et al., 2004; National Resource Center for Academic Detailing, 2013).

### Covariates

Covariates included the following: sex, age group (50–64, 65–79,≧80), geographical area of residence (northern, central, southern, and eastern Taiwan), urbanization level (levels 1 to 4, as described below), monthly income (in New Taiwan dollars (NT$): < 18,000, 18,000–34,999, ≥35,000), and comorbidities. The urbanization of a patient's residence was defined by the population and indicators of the area's level of development. Level 1 urbanization was defined as a population of >1,250,000. Level 2 was defined as a population between 500,000 and 1,250,000. Urbanization levels 3 and 4 were defined as having a population between 150,000 and 500,000 and less than 150,000, respectively. Taiwan's NHI insurance premiums are based on their work salaries. Those with insurance premiums of < NT$18,000 (around US$563) a month are mostly people with lower-than-average monthly incomes or people who are not in the labor force, such as retirees, housewives, or students; those with insurance premiums of NT$18,000–NT$34,999 a month are considered to be in the mean income range (Lin et al., 2013).

In addition, baseline comorbidities with the code ICD-9-CM are listed in **Table S1** with references from the two previous studies (Gray et al., 2015; Richardson et al., 2018). These comorbidities, which were collected at the entry time in the study period in the exposed or unexposed groups, included the following: diabetes mellitus, hypertension, hyperlipidemia, stroke and transient ischemic attack, hemorrhagic stroke, transient ischemic attack, heart failure, peripheral vascular disease, coronary artery disease, atrial fibrillation, angina, myocardial infarction, deep vein thrombosis, Parkinson's disease, epilepsy, fatigue, hemiplegia and paraplegia, headache, tension-type headache, other headache syndromes, back or neck pain, peripheral neuropathy, Meniere's disease, restless leg syndrome, chronic obstructive pulmonary disease, asthma, rhinitis, gastroesophageal reflux disease, peptic or gastric ulcer, irritable bowel syndrome, inflammatory bowel disease, liver disease (except alcoholic liver diseases), osteoarthritis, rheumatoid arthritis, eczema and dermatitis, psoriasis, urinary incontinence, chronic kidney disease, cancer, prostatism, falls, fractures, obesity, depressive disorder, bipolar disorder, anxiety disorder, nonorganic sleep disorder, organic sleep disorder, and psychotic disorder, with references from the two previous studies (Gray et al., 2015; Richardson et al., 2018).

### Outcome Measures

All of the participants enrolled in the present study were followed from the index date until the onset of dementia, withdrawal from the NHI program, death, or the end of 2015. Dementia was identified by the ICD-9-CM codes of 290.0, 290.10, 290.11, 290.12, 290.13, 290.20, 290.21, 290.3, 290.41, 290.42, 290.43, 290.8, 290.9, and 331.0. The types of dementia are grouped as follows: Alzheimer dementia (AD, ICD-9-CM 331.0), vascular dementia (VaD, ICD-9-CM 290.41-290.43), and other dementia (ICD-9-CM 290.0, 290.10-290.13, 290.20-290.21, 290.3, and 290.8-290.9).

A total of 197,560 patients who had used anticholinergics were enrolled (the study cohort), and another 592,680 patients who had not used anticholinergics were enrolled as the non-users (the comparison cohort) (**Figure 1**); thus, there was a relative 1:3 ratio of the anticholinergic cohort and the control cohort. In the anticholinergic usage cohort, 1,818 in 1975,60 individuals (0.92%) were missing due to loss of tracking and 5,394 in 592,680 (0.91%) were loss of tracking in the comparison cohort, during the 15-year of study period.

### Statistical Analysis

All analyses were performed using the SPSS software version 22.0 for Windows (IBM Corp., Armonk, NY). χ^2^ and t-tests were used to evaluate the distributions of the categorical and continuous variables between the patients who did and did not use anticholinergics. The time-dependent Cox regression model was used to determine the risk of dementia, and the results are presented as a hazard ratio (HR) with a 95% confidence interval (CI), censoring with death. The differences in the risk of dementia between the two groups were estimated using the CICR model method, using the computer program as STATS_COMPRISK,spe, with the log-rank test. The HR analyses were for the types, duration of usage, and cognitive burden of the anticholinergic medications. The risk of BPSD was also analyzed, and the HR analyses were for the usage of anticholinergic medications and the usage of psychotropic drugs in the patients. In addition, the interaction tests were conducted to reveal the interactions between age, sex, and comorbidities and the risk of dementia in patients with anticholinergic usage, and the sensitivity analysis was used by excluding the diagnoses of dementia in the first 2 or 4 years, to eliminate any potential protopathic bias. A two-tailed p-value < 0.05 was considered to be statistically significant.

### Ethical Approval

This study was carried out in accordance with the recommendations of the NHI Administration, which has given general approval for the use of their data in this research (Chen et al., 2011).

According to the Declaration of Helsinki (World Medical Association, 2013), The protocol was approved by the Institutional Review Board and the Ethical Committee (IRB/EC) of the Tri-Service General Hospital (IRB No. 2-107-05-026), and the IRB/EC of the Tri-Service General Hospital has exempted the requirement for written informed consents in this study, since any identifiable, personal information included in the NHIRD was encrypted to protect the patient's individual privacy (Chen et al., 2011).

## Results

### Baseline Characteristics of the Study Population

**Table 1** shows the sex, age, comorbidity, level of urbanization, and geographical location of the anticholinergic users and the non-users. The anticholinergic users were associated with lower comorbidity rates of diabetes mellitus, stroke, heart failure, Parkinson's disease, epilepsy, hemiplegia and paraplegia, back or neck pain, Meniere's disease, chronic obstructive pulmonary syndrome, asthma, peptic or gastric ulcer, liver disease, chronic kidney disease, and cancer than the non-users. In contrast, the anticholinergic users were associated with higher comorbidity rates of hypertension, hyperlipidemia, myocardial infarction, headache, rhinitis, a fall injury, a fracture, obesity, depression, bipolar disorder, anxiety, and nonorganic sleep disorders than the non-users. In addition, the patients who used anticholinergics received more hospital-based care.

**Table 1:** Characteristics of study at the baseline.

Anticholinergic medications	With		Without		P
Variables	n	%	n	%	
**Total**	197,560	25.00	592,680	75.00	
**Sex**					0.999
Male	101,059	51.15	303,177	51.15	
Female	96,501	48.85	289,503	48.85	
**Age (years)**	64.32 ± 9.99		64.35 ± 9.89		0.244
**Age group (years)**					0.999
50–64	100,465	50.85	301,395	50.85	
65–79	58,012	29.36	174,036	29.36	
≧80	39,083	19.78	117,249	19.78	
**Education (years)**					0.576
< 12	108,345	54.84	325,632	54.94	
≧12	89,215	45.16	267,048	45.06	
**Insured premium (NT$)**					0.286
< 18,000	164,558	83.30	493,129	83.20	
18,000–34,999	20,090	10.17	61,208	10.33	
≧35,000	12,912	6.54	38,343	6.47	
**Diabetes mellitus**	33,513	16.96	107,557	18.15	<0.001
**Hypertension**	51,933	26.29	131,234	22.14	<0.001
**Hyperlipidemia**	8,869	4.49	18,374	3.10	<0.001
**Stroke**	16,884	8.55	59,213	9.99	<0.001
**Heart failure**	5,238	2.65	20,211	3.41	<0.001
**Peripheral vascular disease**	115	0.06	412	0.07	0.996
**Atrial fibrillation**	3,578	1.81	11,103	1.87	0.186
**Angina**	3,294	1.67	9,788	1.65	0.737
**Myocardial infarction**	6,475	3.28	17,677	2.98	<0.001
**Deep vein thrombosis**	496	0.25	1,325	0.22	0.124
**Parkinson's disease**	1,347	0.68	4,533	0.76	0.006
**Epilepsy**	441	0.22	1,791	0.30	0.001
**Hemiplegia & paraplegia**	2,568	1.30	10,927	1.84	<0.001
**Headaches**	1,024	0.52	2,718	0.46	0.013
**Back or neck pain**	477	0.24	1,831	0.31	0.002
**Peripheral neuropathy**	2,113	1.07	6,755	1.14	0.062
**Meniere's disease**	619	0.31	3,201	0.54	0.001
**Restless leg syndrome**	0	0	0	0	–
**Chronic obstructive pulmonary syndrome**	12,753	6.46	55,060	9.29	<0.001
**Asthma**	4,171	2.11	17,827	3.01	<0.001
**Rhinitis**	1,077	0.55	1,904	0.32	<0.001
**Gastroesophageal reflux**	659	0.33	5	<0.01	<0.001
**Peptic or gastric ulcer**	13,322	6.74	47,185	7.96	<0.001
**Irritable bowel syndrome**	212	0.11	680	0.11	0.998
**Inflammatory bowel disease**	139	0.07	501	0.08	0.184
**Liver disease**	6,378	3.23	35,096	5.92	<0.001
**Osteoarthritis**	6,804	3.44	16,088	2.71	<0.001
**Rheumatoid arthritis**	767	0.39	2,333	0.39	0.865
**Eczema & dermatitis**	1,010	0.51	3,113	0.53	0.682
**Psoriasis**	176	0.09	509	0.09	0.797
**Urinary incontinence**	154	0.08	379	0.06	0.257
**Chronic kidney disease**	6,759	3.42	35,768	6.03	<0.001
**Cancer**	17,149	8.68	55,829	9.42	<0.001
**Prostatism**	0	0	0	0	–
**Falls**	8,180	4.14	20,135	3.40	0.002
**Fractures**	18,795	9.51	44,736	7.55	<0.001
**Obesity**	96	0.05	172	0.03	0.014
**Depression**	1,425	0.72	330	0.06	<0.001
**Bipolar disorder**	201	0.10	469	0.08	0.042
**Anxiety**	1,562	0.79	3,811	0.64	0.002
**Non-organic sleep disorders**	1,939	0.98	4,442	0.75	0.001
**Organic sleep disorders**	3	<0.01	1	<0.001	<0.001
**Psychotic disorders**	1,495	0.76	4,720	0.80	0.297
**Location**					<0.001
Northern Taiwan	77,954	39.46	229,607	38.74	
Middle Taiwan	56,611	28.66	168,415	28.42	
Southern Taiwan	51,434	26.03	154,980	26.15	
Eastern Taiwan	10,674	5.40	36,810	6.21	
Outlets islands	887	0.45	2,868	0.48	
**Urbanization level**					<0.001
1 (The highest)	63,609	32.20	195,570	33.00	
2	89,528	45.32	259,075	43.71	
3	13,068	6.61	42,383	7.15	
4 (The lowest)	31,355	15.87	95,652	16.14	
**Level of care**					<0.001
Medical center	74,815	37.87	212,693	35.89	
Regional hospital	78,602	39.79	203,733	34.37	
Local hospital	44,143	22.34	176,254	29.74	

P, Chi-square test on category variables and t-test on the continuous variables; NT$, New Taiwan Dollars.

### CICR Curves for Dementia in Patients With the Usage of Anticholinergic Medications

**Figure 2:**
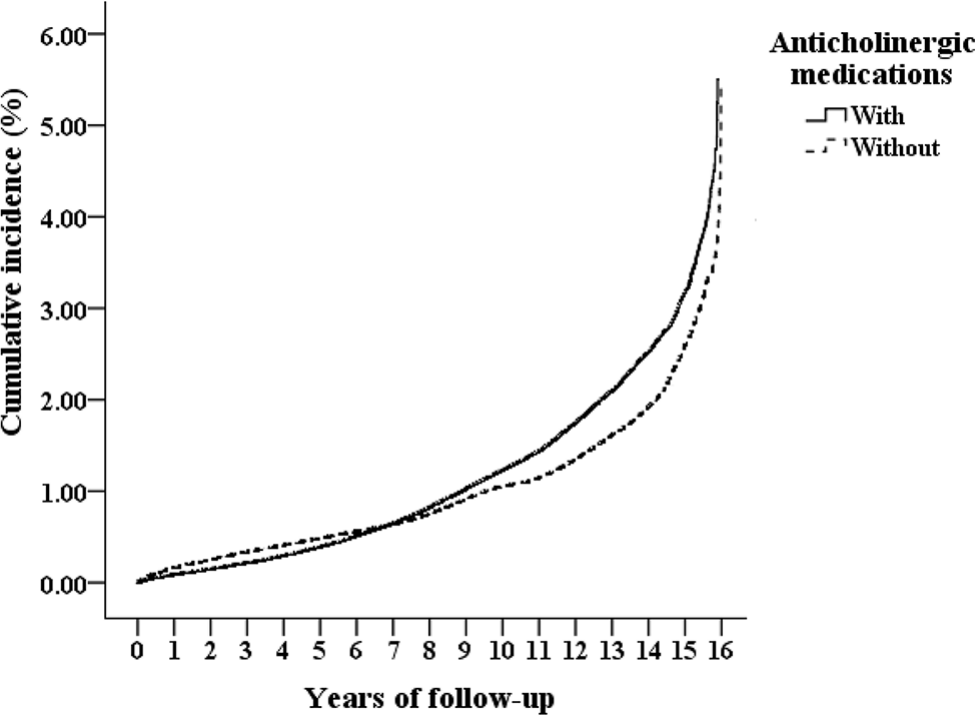
Cumulative incidence competing risk (CICR, the vertical axis) of dementia aged 50 years and over, stratified based on the exposure of anticholinergic medications, in the 15-year of follow up (the horizontal axis), with log-rank test (Log-rank *p =* 0.178).

At the end of the present study, 46,820 of the 790,240 enrolled patients developed dementia, including 11,737 of the 197,560 (523.84 per 100,000 person-years) patients who used anticholinergics and 35,083 of the 592,680 (482.31 per 100,000 person-years) non-users patients (**Figure 1**). **Figure 2** shows the CICR curves, by using the computer program as STATS_COMPRISK,spe, for the CICR of dementia for the study cohort and for the comparison cohort with the log-rank test. There was no difference between the anticholinergic users and the non-users in the risk of development of dementia over the 15-year follow-up period (*p* = 0.178).

### The Usage of Anticholinergic Medications Is Not Associated With an Increased HR for Dementia

**Table 2** shows that the anticholinergic usage was not associated with the increased risk of dementia as the HR was 1.043 (95% CI = 0.958-1.212, *p*  = 0.139). **Table S3** shows that hazard ratios was 1.054 (95% CI  = 0.930-1.175, *p =* 0.088) with the analysis by using only the level 2 and level 3 anticholinergic medications. In this study, most of the comorbidities are as mentioned above, and the locations in Taiwan, urbanization levels, and levels of medical care were not associated with the increased risk of dementia (data not shown). Male patients, patients of age of 65–79 and ≥ 80 years, and comorbidities, such as stroke, Parkinson's disease, epilepsy, hemiplegia and paraplegia, asthma, urinary incontinence, depression, bipolar disorder, and psychotic disorder, were independent risk factors of dementia, in both all levels of ACB (**Table 2**) and ACB of Level 2 and Level 3 rated drugs (**Table S3**). In addition, the interaction test revealed that age male patients and patients of age of 65-79 and ≥ 80 years in the anticholinergic usage cohort were associated with the risk of dementia. However, the association between several comorbidities, such as stroke, hemiplegia and paraplegia, and asthma, and the risk of dementia, were statistically insignificant (**Table 2**).

**Table 2:** Hazard ratios of dementia in different factors, analyzed by using time-dependent Cox regression analysis.

Variables	Time-dependent Cox regression									Interaction test
****	**Crude HR**	**95% CI**	**95% CI**	***P***	**aHR**	**95% CI**	**95% CI**	***P***		***P***
**Anticholinergic medications**										
**Without**	Reference				Reference					
**With**	1.095	0.966	1.225	0.127	1.043	0.958	1.212	0.139		
**Male *(Reference: female)***	1.083	1.015	1.146	0.017	1.019	1.005	1.033	0.045		0.025
**Age 65-79 *(Reference: age 50–64)***	1.085	1.025	1.139	0.026	1.031	1.012	1.056	0.031		0.006
**Age ≧ 80 *(Reference: age 50–64)***	2.201	2.167	2.286	<0.001	1.429	1.400	1.456	<0.001		
**Stroke *(Reference: without)***	3.253	3.172	3.337	<0.001	1.656	1.633	1.680	<0.001		0.058
**Parkinson's disease *(Reference: without)***	4.399	4.200	4.607	<0.001	1.652	1.612	1.690	<0.001		0.002
**Epilepsy *(Reference: without)***	3.745	3.434	4.073	<0.001	1.485	1.423	1.552	<0.001		0.009
**Hemiplegia & paraplegia *(Reference: without)***	2.836	2.626	3.063	<0.001	1.100	1.057	1.147	<0.001		0.235
**Asthma *(Reference: without)***	1.833	1.766	1.904	<0.001	1.102	1.025	1.188	0.022		0.179
**Urinary incontinence *(Reference: without)***	2.419	1.657	2.786	<0.001	1.249	1.095	1.424	<0.001		0.042
**Depression *(Reference: without)***	2.614	2.108	3.243	<0.001	1.410	1.359	1.463	<0.001		<0.001
**Bipolar disorder *(Reference: without)***	2.614	2.108	3.243	<0.001	1.442	1.295	1.609	<0.001		0.001
**Psychotic disorders *(Reference: without)***	2.254	2.059	2.467	<0.001	1.296	1.236	1.355	<0.001		<0.001

HR, hazard ratio, CI, confidence interval, aHR, Adjusted hazard ratio: Adjusted for the variables listed in the table.

T_Cov × Anticholinergic medications: The analysis of the interaction between anticholinergic medications and dementia in different time-periods, the P was 0.007 (Crude HR model), the P was 0.053 (aHR model), after adjusted all the covariates, which is statistically insignificant.

### HR Analyses of the Types, Duration, Cognitive Burden of Anticholinergic Medications, and the Risk of Dementia

In this study, most of the types of the anticholinergic medications, the duration of the anticholinergic usage <1,460 days, and the Anticholinergic Cognitive Burden scale (ACB scale) < 5, were not associated with the risk of dementia. The time-dependent Cox regression hazards risk model showed significant findings in the usage of antiparkinsonian drugs were associated with the risk of dementia, HR: 1.328 [95% CI, 1.038–1.677, *p =* 0.015]. In addition, the longer duration of the anticholinergic exposure ≥1,460 days, HR: 1.191 [95% CI, 1.005–1.386, *p =* 0.045], and a higher level ACB scale scores, ≥5,HR: 1.317 [95% CI, 1.041–1.530, *p =* 0.001], were associated with the risk of dementia (**Table 3**).

**Table 3:** Factors of dementia by using time-dependent Cox regression analysis among different models.

Model	Anticholinergic medications					Time-dependent Cox regression			
****	**Subgroup**	**Events**	**Population**	**PYs**	**Rate (per 10^5^ PYs)**	**aHR**	**95% CI**	**95% CI**	***P***
**Model 1**	Without	34,563	592,680	7,274,012	475.16	Reference			
**With/Without**	With	11,562	197,560	2,240,667	516.01	1.043	0.958	1.212	0.139
**Model 2**	Antidepressants	1,683	13,755	193,180	871.21	1.127	0.926	1.395	0.196
**Types of anticholinergics**	Antipsychotic	2,874	35,121	454,513	632.33	1.108	0.881	1.361	0.213
**(Reference: without)**	Antiparkinsonian drugs	512	1,836	23,444	2,183.93	1.328	1.038	1.677	0.015
	Analgesics	6,274	110,040	1,317,079	476.36	0.997	0.805	1.277	0.633
	Cardiovascular drugs	8,195	98,192	1,300,518	630.13	1.077	0.865	1.434	0.434
	Gastrointestinal drugs	5,198	69,898	907,841	572.57	1.049	0.883	1.270	0.166
	Respiratory drugs	6,471	73,617	965,142	670.47	1.086	0.891	1.146	0.197
	Urological drugs	603	4,542	66,038	913.11	1.129	0.929	1.234	0.135
****	Other drugs	6,210	76,643	1,001,697	619.95	1.048	0.831	1.121	0.304
**Model 3**	0	34,563	592,680	7,274,012	475.16	Reference			
**Anticholinergic medications durations**	1–3 days	1,514	28,442	337,055	449.18	0.995	0.819	1.211	0.397
**(drug exposure period)**	14–89 days	2,006	37,643	437,136	458.90	1.056	0.921	1.240	0.284
	90–364 days	2,391	43,490	488,266	489.69	1.126	0.955	1.293	0.142
	365–1,459 days	2,888	54,292	528,679	546.27	1.158	0.979	1.315	0.079
	≧1,460 days	2,763	33,693	449,531	614.64	1.191	1.005	1.386	0.045
**Model 4**	0	34,563	592,680	7,274,012	475.16	Reference			
**Anticholinergic Cognitive Burden scale**	<1	1,865	36,654	398,053	468.53	1.032	0.759	1.044	0.597
	1–1.9	2,297	42,781	475,926	482.64	1.042	0.848	1.084	0.422
	2–2.9	3,432	52,222	697,301	492.18	1.132	0.935	1.291	0.126
	3–4.9	1,981	34,820	352,483	562.01	1.206	0.990	1.359	0.056
	≧5	1,987	31,083	316,904	627.00	1.317	1.041	1.530	0.001

PYs, Person-years; aHR, Adjusted hazard ratio: Adjusted for the variables listed in **Table 1**; CI, confidence interval; P, Chi-square test on category variables and t-test on the continuous variables.

### A Sensitivity Analysis for the Association Between the Usage of Anticholinergic Medications and Dementia

We have conducted the sensitivity analysis to evaluate the risk of dementia. We have excluded the patients diagnosed with dementia within the first 2 and the first 4 years after the usage of anticholinergics. No association between anticholinergic usage was found after excluding the diagnosis of dementia with the first 2 and the first 4 years after the usage of anticholinergics (**Table 4**).

**Table 4:** Factors of dementia subgroup and sensitivity analysis by using time-dependent Cox regression analysis.

Anticholinergic medications (With vs. Without)		Time-dependent Cox regression			
**Sensitivity analysis**	**Dementia subgroup**	**aHR**	**95% CI**	**95% CI**	***P***
**Overall**	All dementia	1.043	0.958	1.212	0.139
	AD	1.115	0.990	1.296	0.060
	VaD	1.039	0.975	1.208	0.149
****	Other degenerative dementia	1.031	0.964	1.207	0.178
**First 2 years excluded**	All dementia	1.040	0.977	1.208	0.089
	AD	1.112	0.987	1.290	0.075
	VaD	1.023	0.961	1.188	0.224
****	Other degenerative dementia	1.036	0.973	1.205	0.138
**First 4 years excluded**	All dementia	1.024	0.962	1.190	0.162
	AD	1.087	0.982	1.263	0.077
	VaD	0.994	0.934	1.155	0.394
	Other degenerative dementia	1.023	0.961	1.189	0.248

PYs, Person-years; aHR, Adjusted hazard ratio: Adjusted for the variables listed in **Table 1**.; CI, confidence interval; AD, Alzheimer dementia; VaD, Vascular dementia; P, Chi-square test on category variables and t-test on the continuous variables.

### HR Analysis for the Usage of Anticholinergic Medications and the Usage of Psychotropic Drugs

There were no direct codes in the ICD-9-CM system for the BPSD, however, we identified a substitute for the BPSD by recording the usage of psychotropics, such as antipsychotics, antidepressants, and hypnotics after the dementia diagnosis in the anticholinergic cohort or the control group. **Table 5** shows that in the patients who developed dementia during this study, there were no significant differences in the usage of overall psychotropic drugs, antipsychotics, and antidepressants between the patients from the anticholinergic cohort and the non-users. The only exception was the marginal difference between the usage of hypnotics in the anticholinergic cohort and the non-users as follows: 40.03% (N = 79,083) vs 38.44% (N = 227,855), respectively, (*p =* 0.015). In addition, there were no differences between the usage of combination drugs, monotherapy drugs, or none of the psychotropic drugs in these two groups.

**Table 5:** Usage of psychotropic drugs in the dementia patients.

Dementia	With anticholinergics (N = 197,560)	Without anticholinergics (N = 592,680)	P
**Overall psychotropic drugs**	188,927 (95.63%)	564,409 (95.23%)	0.196
**Antipsychotics**	103,028 (52.15%)	306,239 (51.67%)	0.484
**Antidepressants**	89,176 (45.14%)	271,625 (45.83%)	0.322
**Hypnotics**	79,083 (40.03%)	227,855 (38.44%)	0.015
**Combination of different classes of drugs**			0.346
**≧2**	73,354 (37.13%)	216,862 (36.59%)	
**1**	115,572 (58.50%)	347,547 (58.64%)	
**0**	8,634 (4.37%)	28,271 (4.77%)	

P, Chi-square test on category variables and t-test on the continuous variables.

## Discussion

### No Association Exists Between the Usage of Anticholinergic Medications and the Risk of Dementia

In this 15-year follow-up cohort study, the usage of anticholinergic medications was not associated with the risk of dementia. At the end of the study, the rate of patients with dementia was 5.92%, which is within the range of the finding of 4%–8% in the previous community studies on dementia prevalence in Taiwan in those aged ≥65 years (Liu et al., 1995; Liu et al., 1998a; Liu et al., 1998b; Sun et al., 2014); thus confirming that this study was conducted on a representative sample population.

Protopathic bias arises when the initiation of a drug (exposure) occurs in response to a symptom of the (at this point undiagnosed) disease under study (outcome), which reflects a reversal of cause and effect. This is particularly a problem in studies of drug-cancer associations and other outcomes with long latencies We handled this bias by the sensitivity analysis (Gerhard, 2008; European Network of Centres for Pharmacoepidemiology and Pharmacovigilance, 2018). Based on the sensitivity analysis in **Table 4** and the year-tracking comparison between anticholinergic users and no-users in "supplementary-material" rid="SM **Table S2**, the usage of anticholinergic medications was not found to be associated with overall dementia. Also, when protopathic bias and the carry-over effects (Schuemie et al., 2012) were removed by excluding the first two and four years of dementia, there was no statistical significance. To the best of our knowledge, this is the first nationwide, population-based, matched-cohort study with a 15-year follow-up on the topic of the association between the usage of anticholinergic medications and the risk of dementia. The results from this study are different from the findings of previous studies, which revealed the association between the usage of anticholinergic medications and the increased risk of dementia (Carriere et al., 2009; Jessen et al., 2010; Gray et al., 2015; Richardson et al., 2018).

### Comparison of This Study With the Previous Literature

Two cohort studies (Jessen et al., 2010; Gray et al., 2015) and two case control studies (Carriere et al., 2009; Richardson et al., 2018) have reported the association between anticholinergic medication usage and the risk of dementia. In comparison to these studies, our study has several advantages: First, it was conducted in a nationwide, population-based sample, instead of in regional samples like the other studies. Second, we used a larger sample size of approximately 400,000 patients, which outnumbered those of other studies. Third, our study spanned a 15-year follow-up, longer than the other studies with either a 4-year (Jessen et al., 2010) or 10-year follow-up period (Gray et al., 2015).

In the present study, the risk of dementia was found to be increased with a longer duration of anticholinergic usage (≥1,460 days) or a higher ACB scores (a score of 5 or higher), and these results were similar to the findings of Gray and colleagues (2015) (≥1,095 days) (Richardson et al., 2018) and Richardson and colleagues (2018) (Gray et al., 2015). In addition, cardiovascular drugs, analgesics, and respiratory drugs were the three leading medications with anticholinergic effects in the present study. It is highly possible that it is the physical problems themselves, or certain comorbidities that increase with aging, which needed to be treated with anticholinergic medications, were responsible for the risk of dementia. Thus, we might design a multicenter prospective cohort study, to include different indications and stratify by different indications. This may allow to conclusively show that the results are caused by the exposure rather than the indication (Catalogue of bias collaboration, et al., 2018). In addition, there was an apparent “dose-response effect” with the increased risk for higher ACB, and longer duration of anticholinergic exposure; however, the medical conditions that led the patients to take the anticholinergic medications might also contribute to the risk factors for dementia. Nonetheless, this observation should remind clinicians to be cautious when prescribing medications with a high ACB score and a longer duration of anticholinergic usage.

A similar finding in a recent nested case control study found that the anticholinergic antidepressants, antiparkinsonian drugs, antipsychotics, bladder antimuscarinic drugs, and antiepileptic drugs were associated with the risk of dementia. All anticholinergic medications with longer duration of the usage for more than 1,095 total standardized daily doses (TSDDs) were associated this risk. These associations were stronger in cases diagnosed before the age of 80 years (Coupland et al., 2019).

### Sex and the Risk of Dementia

In this study, male sex was associated with risk of dementia. Previous studies have shown that, in AD, low education has been historically a risk factor for women, bilateral oophorectomy is a factor associated with women, and the apolipoprotein E genotype is equally common in men and women but has a stronger effect in women (Rocca et al., 2014). On the other hand, male patients with Parkinson's disease progressed more rapidly than females in the transition from no cognitive impairment to Parkinson's disease dementia (Cholerton et al., 2018). However, the influences of sex on the risk of dementia remains unclear and further studies may well be needed to examine the association among male patients, anticholinergic usage, and the risk of dementia.

### Aging, Comorbidities and the Risk of Dementia

In our study, the patients with anticholinergic exposure aged 65–79 and ≥80 years were associated with a higher risk of dementia, in comparison to the patients with aged 50–64 years. Several previous studies have reported that aging itself is a risk factor of dementia development (Liu et al., 1995; Liu et al., 1998a; Liu et al., 1998b; Sun et al., 2014).

Along with previous evidence, our data also demonstrate that, in certain patient groups with several comorbidities, anticholinergics treatment had associations with the risk of dementia. These comorbidities themselves may serve as independent risk factors for dementia, including stroke (Yang et al., 2015b; Corraini et al., 2017; Shih et al., 2017), Parkinson's disease (Breteler et al., 1995; Riedel et al., 2008; Jessen et al., 2010), epilepsy (Breteler et al., 1995; Sen et al., 2018), hemiplegia and paraplegia (Bejot et al., 2011; Huang et al., 2017), asthma (Rusanen et al., 2013; Su et al., 2014; Peng et al., 2015), depression (Yang et al., 2015b; Yang et al., 2015c; Huang et al., 2017), bipolar disorder (Wu et al., 2013; Yang et al., 2015b), and psychotic disorder (Lin et al., 2018a; Almeida et al., 2019). Notice that urinary incontinence was one of the key components of impaired activity daily living (ADL) (Gosch et al., 2015; Greer et al., 2015), which is a predictor of the risk of dementia (Fauth et al., 2013). However, the association between several comorbidities, such as stroke, hemiplegia and paraplegia, and asthma, and the risk of dementia, were statistically insignificant in this study.

Overall, we might well attribute the risk of dementia to aging and the comorbidities themselves, instead of the impact from the usage of anticholinergic medications, per se. Since anticholinergic usage could be related to drowsiness, delirium, sedation, and memory problems (Ruxton et al., 2015), especially among frail, older patients (Tannenbaum et al., 2012), clinicians are advised to balance the risks against benefits when considering the usage of anticholinergic medications for the elderly.

### The Different Types of Anticholinergic Medications and Their Impact on Cognitive Function

In this study, the usage of antiparkinsonian anticholinergics was associated with the increased risk of dementia. Antiparkinsonian drugs were mostly used for Parkinson's disease (Leoni et al., 2002; De Germay et al., 2016) and for managing the extrapyramidal side effects with antipsychotic usage for psychotic disorders (Hunter, 1981; Xiang et al., 2007). Therefore, this finding might also be related to the fact that Parkinson's disease and psychotic disorders have been associated with the risk of dementia as aforementioned. Several previous studies have reported the association between antidepressant usage and the risk factor for dementia (Lee et al., 2016; Then et al., 2017); nevertheless, our study revealed no such association between antidepressants with anticholinergic effects (most tricyclic antidepressants) and the risk of dementia. In addition, one study using the NHIRD database reported the association between antimuscarinics for an overactive bladder (OAB) and the subsequent dementia risk in patients with diabetes mellitus (Yang et al., 2017). However, in our study, the usage of urological drugs (antimuscarinics, such as oxybutynin, solifenacin, and tolterodine) was not associated with the risk of dementia. In addition, a recent study found that OAB, instead of the use of antimuscarinic urological drugs, was associated with the increased risk of psychiatric disorders, including dementia (Tzeng et al., 2019a).

### The Use of Anticholinergic Medications and BPSD

It is needed to appraise the influences of anticholinergic usage on BPSD, since the exposure to some medications could result in long term effects. For example, antipsychotics might cause long-term effects such as tardive dyskinesia. Antipsychotics and anticholinergic might share some mechanisms, which were underscored by the proposed use of anticholinergics to treat tardive dyskinesia (Bergman and Soares-Weiser, 2018). For collecting the data of long-term side effects, the post-marketing pharmacovigilance is necessary regarding newer muscarinic antagonists, such as glycopyrronium, aclidinium, and umeclidinium (Tashkin, 2015), since the anticholinergic drugs are related to the side effects as tooth decay, cardiac side effects (Rogliani et al., 2018), and the increased risk of cancer in patients with chronic obstructive pulmonary disease (COPD) (Lin et al., 2018b).

Furthermore, while we recognized BPSD from the records of usage of psychotropics, such as antipsychotics, antidepressants, and hypnotics, after dementia was diagnosed in the anticholinergic cohort or the non-users, there were no significant differences between the anticholinergic and the comparison cohorts in the overall usage of the psychotropic drugs, antipsychotic drugs, and antidepressants in this study; in addition, there was quite a marginal difference between the usage of hypnotics at 40.03% vs 38.44% (*p =* 0.015) in the anticholinergic cohort and the non-users, respectively. Monotherapy or polypharmacy showed no significant difference between the anticholinergic usage and psychotropic usage in both groups. In other words, the anticholinergic drugs were not associated with BPSD, which was represented by the usage of the psychotropic medications after the diagnosis of dementia. Antiepileptic medications were not included in this analysis since antiepileptics, such as carbamazepine and valproic acid, had limited or conflicting effects for the treatment of BPSD (Sink et al., 2005; Franco and Messinger-Rapport, 2006; Yeh and Ouyang, 2012; Tzeng et al., 2017b), and one previous study in Taiwan has shown that only 1% of the patients with BPSD received antiepileptics as a treatment (Tsai et al., 2010). A multicenter, prospective follow-up study might be necessary to clarify the association between the previous use of anticholinergic medications and the risk of BPSD.

The reason why the anticholinergic usage was not associated with the risk of BPSD remains unclear. We hypothesize that the upregulation of muscarinic receptors, in the long-term use of anticholinergic medications (Hori et al., 2014), might play a role in such a phenomenon. However, more studies are needed to clarify the underlying mechanisms.

### Limitations

Several limitations/concerns of the present study should be addressed. First, as in many previous NHIRD-based studies, our study is retrospective and dependent upon the ICD-9-CM codes instead of the direct medical records or the interview data. It is necessary to conduct a study, with a prospective cohort study design, to achieve a more accurate and consistent finding. Therefore, the lack of detailed records and misdiagnosis-related errors may have occurred. Second, the NHIRD database does not contain genetic, nutritional, or habitual factors, such as the apolipoprotein E genotype, record of smoking, and body mass index that were not included in this database, and in such a claims database study, we could only estimate the treatment durations of each anticholinergics by dividing the cumulative doses of individual medications by DDD. The role of blood brain barrier (BBB) plays an important role in the central nervous system side effects of anticholinergic medications. The factors determining the penetration of anticholinergic medications might include passive diffusion, active transport, lipophilicity, the polarity of the chemical, and molecular size (Staskin and Zoltan, 2007). Small, lipophilic, noncharged molecular compounds (tertiary ammonium groups) pass the BBB more readily than those containing a quaternary ammonium group (van de Waterbeemd et al., 1998). Furthermore, the data such as serial amyloid scans or some more sensitive measure of dementia were not available in this NHIRD claims database. Third, this national review insurance database cannot provide detailed information, including the severity, stage, and care-giver burden of the patients with dementia. Fourth, the use of the ACB scale has some limitations as it includes drugs without clinically relevant negative cognitive effects, and there is a lack of serum anticholinergic activity levels validation. Fifth, the recognition of BPSD by the records of psychotropic usage is also a limitation. However, in one study in Taiwan, only 7% of patients with dementia did not receive any psychiatric medication (Lin, 2011). Sixth, the records of the medications obtained from the NHIRD for big data studies, including the anticholinergic study, were listed as the total dose(s) of the referred medication(s) in this database. Therefore, we identified the use of the patients who first started an anticholinergic regimen between January 1, 2000 and December 31, 2015, instead of the exact date of anticholinergic usage. Seventh, it is not easy to distinguish the effects of anticholinergic drugs, and know the underlying disease and permeability of crossing the blood brain barrier types of anticholinergics in the present study. Seventh, since the nonusers were individual who never used an anticholinergic during the 15-year period, there could be a limitation producing the conditioning on future exposure and risk of bias (Lund et al., 2017). Finally, the timing of the records of the use of psychotropics (i.e., antipsychotics, antidepressants, and hypnotics) to recognize BPSD could not be identified, since these were also listed as the total dose(s) of the referred medication(s) in this database. The survival bias might occur by using this method of estimation of duration of the anticholinergic usage to investigate the risk of dementia, with the reference from one previous study of a similar design (Gray et al., 2015). However, we used the time-varying cumulative exposure analysis, to avoid the survival bias possible. Therefore, a multicenter, prospective observational study might well be necessary to clarify the association between the previous use of anticholinergic medications and the risk of BPSD.

## Conclusion

The strength of the present study lies in the large population dataset with strong attempts to control the disease-related protopathic bias. In this study, the usage of anticholinergics was not associated with the risk of dementia or BPSD from a 15-year follow-up study in Taiwan. However, the groups of male patients, patients of age of 65–79 and ≥ 80 years, with comorbidities as Parkinson's disease, epilepsy, urinary incontinence, depression, bipolar disorder, and psychotic disorder, higher ACB scores, and the long duration of treatment, were associated with the risk of dementia. Based on the results of this study, clinicians are advised to balance the risks and benefits when considering the usage of anticholinergic medications.

## Ethics Statement

In comparison to the Code of Ethics of the World Medical Association (Declaration of Helsinki) (World Medical Association, 2013), a written informed consent was not obtained from the participants in the encrypted data for this study. Since the identifiable database of the individuals included in the NHIRD were all encrypted in order to protect individual privacy (Chen et al., 2011), the NHI Administration has given general approval for their data to be used in this research (Chen et al., 2011). Because the NHIRD has the advantage in providing a large-scale, longitudinal, reliable dataset, leading to extensive uses for the population-based researches in Taiwan (Hsiao et al., 2007; Chen et al., 2011; National Health Research Institutes, 2015), the Institutional Review Board (IRB, the ethical committee) of the Tri-Service General Hospital was aware of this and approved the research to proceed, and also agreed that the benefit justified waiving the need for individual written informed consent in such a study (IRB No. 2-107-05-026).

## Author Contributions

Y-PL and N-ST conceived and conducted this study. Y-PL, W-CC, N-ST, and C-HC conducted data collection, data analysis, and data interpretation. H-AC and Y-CK conducted data interpretation. Y-PL and N-ST wrote the manuscript. All authors approved this manuscript.

## Funding

This study was funded by the Tri-Service General Hospital Research Foundation (TSGH-C105-130, TSGH-C106-002, TSGH-C106-106, TSGH-C107-004, TSGH-C108-003, and TSGH-C108-151), and the Medical Affairs Bureau, Ministry of Defense of Taiwan (MAB-107-084).

## Conflict of Interest

The authors declare that the research was conducted in the absence of any commercial or financial relationships that could be construed as a potential conflict of interest.
