# Supplementary material for: Are Anticholinergic Medications Associated With Increased Risk of Dementia and Behavioral and Psychological Symptoms of Dementia? A Nationwide 15-Year Follow-Up Cohort Study in Taiwan
Source: Front Pharmacol. 2020 Feb 14;11:30. doi: 10.3389/fphar.2020.00030 (PMC7033580; doi:10.3389/fphar.2020.00030)
Supplement: Supplementary file 1 [file Table_1.docx]

| **Table S1. ICD-9-CM codes for the Comorbidity** | |  |
| --- | --- | --- |
| **Comorbidity** | **ICD-9-CM codes** |  |
| **Diabetes mellitus (DM)** | 250 |  |
| **Hypertension (HTN)** | 401.1, 401.9, 402.10, 402.90, 404.10, 404.90, 405.1, 405.9 | |
| **Hyperlipidemia** | 272 |  |
| **Stroke** | 430-436 | |
| **Heart failure (HF)** | 428 |  |
| **Peripheral vascular disease (PVD)** | 443 |  |
| **Atrial fibrillation (AF)** | 427.31 | |
| **Angina** | 413 |  |
| **Myocardial infarction (MI)** | 410-412 | |
| **Deep vein thrombosis (DVT)** | 453 |  |
| **Parkinson's disease** | 332 |  |
| **Epilepsy** | 345 |  |
| **Hemiplegia & paraplegia** | 342, 344 | |
| **Headaches** | 307.81, 339, 346, 784.0 | |
| **Back or neck pain** | 723.1, 724.2 | |
| **Peripheral neuropathy** | 350-358 | |
| **Meniere's disease** | 386.0 | |
| **Restless leg syndrome (RLS)** | 333.94 | |
| **Chronic obstructive pulmonary disease (COPD)** | 490-496 | |
| **Asthma** | 493 |  |
| **Rhinitis** | 472.0, 477 | |
| **Gastroesophageal reflux disease (GERD)** | 530.81 | |
| **Peptic or gastric ulcer** | 531-534 | |
| **Irritable bowel syndrome (IBS)** | 564.1 | |
| **Inflammatory bowel disease (IBD)** | 555-556 | |
| **Liver disease** | 571, except 571.0-571.3 | |
| **Osteoarthritis** | 715 |  |
| **Rheumatoid arthritis (RA)** | 714.0 | |
| **Eczema & dermatitis** | 691-693 | |
| **Psoriasis** | 696.0-696.1 | |
| **Urinary incontinence** | 788.3 | |
| **Chronic kidney disease (CKD)** | 274.1, 403-404, 440.1, 442.1, 447.3, 572.4, 580-589, 642.1, 646.2, 753 | |
| **Cancer** | 140-208 | |
| **Prostatism** | 600.90 | |
| **Falls** | E880-E888 | |
| **Fractures** | 800-829 | |
| **Obesity** | 278 |  |
| **Depression** | 296.2-296.3, 300.4, 311 | |
| **Bipolar disorder** | 296.0-296.1, 296.4-296.9 | |
| **Anxiety** | 300, expect 300.4 | |
| **Non-organic sleep disorders** | 307.4, 780.5 | |
| **Organic sleep disorders** | 327 |  |
| **Psychotic disorders** | 295, 297-298 |  |

**ICD-9-CM: International Classification of Disease, 9th revision, Clinical Modification**
