# Supplementary material for: Are Anticholinergic Medications Associated With Increased Risk of Dementia and Behavioral and Psychological Symptoms of Dementia? A Nationwide 15-Year Follow-Up Cohort Study in Taiwan
Source: Front Pharmacol. 2020 Feb 14;11:30. doi: 10.3389/fphar.2020.00030 (PMC7033580; doi:10.3389/fphar.2020.00030)
Supplement: Supplementary file 2 [file Table_2.docx]

**Table S2. Comparison of cumulative incidence of dementia in patients with or without anticholinergic medications treatment in the tracking years**

| **Anticholinergic medications** | **With** (N = 197,560) | **Without** (N = 592,680) | ***P*** |
| --- | --- | --- | --- |
| **In the tracking of x year(s)** | **Numbers of dementia** | |  |
| **1** | 4,102 | 11,977 | 0.511 |
| **2** | 5,227 | 13,401 | 0.486 |
| **3** | 5,951 | 14,543 | 0.601 |
| **4** | 6,985 | 15,287 | 0.724 |
| **5** | 7,312 | 16,009 | 0.833 |
| **6** | 8,111 | 17,533 | 0.876 |
| **7** | 8,764 | 18,101 | 0.939 |
| **8** | 9,008 | 19,742 | 0.842 |
| **9** | 9,295 | 20,875 | 0.736 |
| **10** | 9,643 | 22,001 | 0.689 |
| **11** | 9,988 | 23,976 | 0.618 |
| **12** | 10,014 | 25,088 | 0.547 |
| **13** | 10,297 | 27,642 | 0.412 |
| **14** | 10,598 | 29,501 | 0.389 |
| **15** | 11,274 | 31,086 | 0.267 |
| **16** | 11,562 | 34,563 | 0.184 |
| ***P:* Chi-square test on category variables and t-test on the continue variables** | | | |
