# Supplementary material for: Are Anticholinergic Medications Associated With Increased Risk of Dementia and Behavioral and Psychological Symptoms of Dementia? A Nationwide 15-Year Follow-Up Cohort Study in Taiwan
Source: Front Pharmacol. 2020 Feb 14;11:30. doi: 10.3389/fphar.2020.00030 (PMC7033580; doi:10.3389/fphar.2020.00030)
Supplement: Supplementary file 3 [file Table_3.docx]

**Table S3. Hazard ratios of dementia for different factors, analyzed by using time-dependent Cox regression analysis among users of level 2 and level 3 rated drugs**

|  | **Time-dependent Cox regression** | | | | | | | |
| --- | --- | --- | --- | --- | --- | --- | --- | --- |
| **Variables** | **Crude HR** | **95% CI** | **95% CI** | ***P*** | **aHR** | **95% CI** | **95% CI** | ***P*** |
| **Anticholinergic medications** |  |  |  |  |  |  |  |  |
| **Without** | Reference |  |  |  | Reference |  |  |  |
| **With** | 1.131 | 0.895 | 1.327 | 0.224 | 1.054 | 0.930 | 1.175 | 0.088 |
| **Male *(Reference: female)*** | 1.109 | 1.131 | 1.167 | <0.001 | 1.044 | 1.041 | 1.083 | 0.003 |
| **Age 60-64 *(Reference: age 50-64)*** | 1.081 | 1.041 | 1.107 | 0.003 | 1.041 | 1.019 | 1.077 | 0.033 |
| **Age ≧ 75 *(Reference: age 50-64)*** | 1.481 | 1.473 | 1.538 | <0.001 | 1.448 | 1.409 | 1.506 | <0.001 |
| **Stroke *(Reference: without)*** | 1.792 | 1.752 | 1.855 | <0.001 | 1.663 | 1.643 | 1.691 | <0.001 |
| **Parkinson's disease *(Reference: without)*** | 1.986 | 1.933 | 2.041 | <0.001 | 1.662 | 1.622 | 1.712 | <0.001 |
| **Epilepsy *(Reference: without)*** | 1.947 | 1.862 | 2.055 | <0.001 | 1.493 | 1.439 | 1.567 | <0.001 |
| **Hemiplegia & paraplegia *(Reference: without)*** | 1.615 | 1.539 | 1.692 | <0.001 | 1.175 | 1.072 | 1.294 | <0.001 |
| **Asthma *(Reference: without)*** | 1.243 | 1.138 | 1.397 | <0.001 | 1.126 | 1.044 | 1.200 | 0.001 |
| **Urinary incontinence *(Reference: without)*** | 1.361 | 1.186 | 1.601 | <0.001 | 1.257 | 1.102 | 1.434 | <0.001 |
| **Depression *(Reference: without)*** | 1.581 | 1.514 | 1.661 | <0.001 | 1.448 | 1.369 | 1.474 | <0.001 |
| **Bipolar disorder *(Reference: without)*** | 1.521 | 1.368 | 1.755 | <0.001 | 1.482 | 1.304 | 1.628 | <0.001 |
| **Psychotic disorders *(Reference: without)*** | 1.426 | 1.357 | 1.526 | <0.001 | 1.305 | 1.252 | 1.369 | <0.001 |

**HR= hazard ratio, CI = confidence interval, aHR = Adjusted hazard ratio: Adjusted for the variables listed in the table**

**T_Cov × Anticholinergic medications: The analysis of the interaction between anticholinergic medications and dementia in different time-periods**

***P:* Chi-square test on category variables and t-test on the continue variables**

***P* = 0.009 (Crude HR model). *P* = 0.077 (aHR model)**
